# Mr. Hutchinson on Leprosy

**Published:** 1903-04-18

**Authors:** 


### The Hospital.

A Journal of

The Medical Sciences and Hospital Administration.

Edited by Sir Henry Burdett, K.C.B.

Vol. XXXIV.—No. 864. [April 18, 1903.

### Mr. Hutchinson on Leprosy.

Whatever may be thought of the evidence against or in favour of Mr. Jonathan Hutchinson's hypothesis that the originating cause of leprosy is the consumption as food of decayed or badly cured fish, there can be no two opinions with regard to his earnest truth-seeking in the matter, or with regard to the energy which he has displayed in his search after any facts which may either confirm or shake his belief. Mr. Hutchinson has for so many years occupied a position of eminence in his profession that we may, without offence, speak of him as no longer young; and yet, at a period of life at which most men seek repose, he has not only made voyages to South Africa and to India for the purpose of investigating the causes of leprosy, but he has also made long and fatiguing journeys into the interior of both countries, entirely on his own responsibility, and at his own expense, and has hunted for facts wherever they could be found with an earnestness that nothing could baffle and a penetration that nothing could deceive. We trust that Mr. Hutchinson will receive the reward for his exertions which he would most appreciate, or, in other words, that he may see the truth of the matter as completely established as is now the truth with regard to the conveyance of intermittent fever by mosquitoes. Mr. Hutchinson belongs to that order of philosophers who love truth better than their system, and therefore, although we think such a result highly probable of attainment, we will not even wish for his sake that he may see his own hypothesis confirmed. Whenever the time comes for the crystallisation of hypothesis into theory we are certain, whatever the theory may be, that he will accept it as a generalised expression of the facts of the case, and that he will regard with unmixed satisfaction the settlement of a point so important to large sections of the human race, and on which views so confused and so contradictory have been held by many generations of physicians.

It is well known that at various times the most diverse opinions have been entertained and expressed with regard to the contagiousness of leprosy; and that at many periods of history and in many countries, the greatest fear of contact with the sufferers has been felt. There are, even now, old churches in England in which "the leper's squint" still remains; that is to say, an obliquely directed aperture or channel through the outer wall, and commanding a view of the altar, through which any leprous parishioner might witness the elevation of the host without being permitted to join the congregation. In many times and at many places the fear of contagion has led the healthy to treat lepers with great cruelty; but the general experience of modern times has led most communities to realise that the fear was very much in excess of the danger. In England, where there are always a few lepers, the nature of the disease is hardly recognised, except by the medical attendants of the patients, and its spreading, even to members of the same family, is utterly unknown. But during his inquiries in South Africa, Mr. Hutchinson arrived at the conclusion that such spreading is liable to occur amongst the members of uncleanly communities, and that it is brought about, in his belief exclusively, by the contamination, by leprous hands, of the food that is eaten by other members of the family of which the leper forms part. This, which he describes as "commensal communication," he regards as practically the only way in which the disease is liable to be conveyed from one person to another, or to occur independently of the consumption of the badly cured fish which he regards as the natural home of the leprous bacillus. It follows that he also regards the buccal or intestinal mucous membranes as the only channels through which this bacillus can find entrance into the human subject.

Mr. Hutchinson's journey to the leper asylums of India, from which he has just returned, and of which an interesting account appeared in the *Times* for the 11th instant, was undertaken mainly in response to the assertion that the natives were not fish eaters, and that hence, with regard to them at least, his hypothesis broke down. In relation to this matter, he has brought back abundant evidence not only that fish is largely eaten, but also that the sections of the community which eat it most largely are precisely those which furnish the largest number of lepers. The most striking illustration of this was afforded by the case of the "Salsette Christians," who are the descendants of converts to Romanism made by the Jesuits under the Portuguese dominion, who inhabit the fishing island of Salsette, adjacent to Bombay, who are fishermen and fish-dealers, who keep the fish fast-days of their church, and who, being only about fifty thousand in number, furnished 369 lepers to the Matunga asylum, against 324 furnished by the whole of the enormous Mussulman population of the Bombay Presidency. In relation to the trustworthiness of commonly-received statements as to fish-eating, Mr. Hutchinson mentions that at one Indian asylum, which he good-naturedly does not name, the native medical officer had prepared for him a return from which it appeared that 40 of the inmates denied having ever eaten fish. Mr. Hutchinson had the 40 brought before him, and questioned them one by one, with the result that only one persisted in the original denial. All the rest confessed to having eaten fish, and said either that they had mistaken the question addressed to them, or else that the doctor had mistaken their reply. The one man who persisted admitted that his parents and friends ate fish habitually, but said that he had not done so because he did not like it. His dislike seems to afford some probability to the supposition that, at some time of his life, he had at least tasted it. It is highly satisfactory to add that leprosy is much less prevalent in India than common rumour has led people to suppose. Mr. Hutchinson reports the prevalence, taking the country as a whole, to be about the same as in Norway, and adds that there has for some years been a steady and continuous decrease.

